# Muscarinic and Nicotinic Contribution to Contrast Sensitivity of Macaque Area V1 Neurons

**DOI:** 10.3389/fncir.2017.00106

**Published:** 2017-12-19

**Authors:** Jose L. Herrero, Marc A. Gieselmann, Alexander Thiele

**Affiliations:** Institute of Neuroscience, Newcastle University, Newcastle upon Tyne, United Kingdom

**Keywords:** acetylcholine, visual cortex organization, contrast sensitivity, normalization, primary visual cortex (V1)

## Abstract

Acetylcholine is a neuromodulator that shapes information processing in different cortical and subcortical areas. Cell type and location specific cholinergic receptor distributions suggest that acetylcholine in macaque striate cortex should boost feed-forward driven activity, while also reducing population excitability by increasing inhibitory tone. Studies using cholinergic agonists in anesthetized primate V1 have yielded conflicting evidence for such a proposal. Here we investigated how muscarinic or nicotinic receptor blockade affect neuronal excitability and contrast response functions in awake macaque area V1. Muscarinic or nicotinic receptor blockade caused reduced activity for all contrasts tested, without affecting the contrast where neurons reach their half maximal response (c50). The activity reduction upon muscarinic and nicotinic blockade resulted in reduced neuronal contrast sensitivity, as assessed through neurometric functions. In the majority of cells receptor blockade was best described by a response gain model (a multiplicative scaling of responses), indicating that ACh is involved in signal enhancement, not saliency filtering in macaque V1.

## Introduction

Acetylcholine (ACh) contributes to sensory processing and to high level cognitive functions across cortical areas. Its action in the primate is probably best studied in primary visual cortex (V1). In V1, ACh application increases neuronal orientation and direction selectivity (Sillito and Kemp, 1983; Sato et al., 1987; Sillito and Murphy, 1987), it increases contrast sensitivity in layer IV by acting on nicotinic receptors (Disney et al., 2007), sharpens spatial tuning (Roberts et al., 2005), reduces firing rate variability and reduces neuronal noise correlations (Goard and Dan, 2009). Moreover, it increases attentional modulation (Herrero et al., 2008), population coding abilities (Minces et al., 2017; van Kempen et al., 2017), and it improves behavioral performance (Pinto et al., 2013; Gritton et al., 2016).

In light of this literature, it is surprising that some of the basic effects of cholinergic activation on neuronal firing rates remain disputed (Bhattacharyya et al., 2012; Disney et al., 2012; Soma et al., 2012). Disney et al. (2007, 2012) report overall decreased activation of macaque V1 neurons upon ACh application outside of the thalamo-recipient layers, and increased activation in thalamo-recipient layers. Conversely, Soma et al. (2012) report an overall facilitatory effect of ACh application in macaque V1 neurons across cortical layers, which was predominantly mediated by muscarinic receptors. The latter is similar to data from the tree shrew, where nicotinic and muscarinic activation increases neuronal activity across all layers, even if overall magnitude differences exist between layers (Bhattacharyya et al., 2012).

All of these studies (Bhattacharyya et al., 2012; Disney et al., 2012; Soma et al., 2012) assessed contrast response function, through application of non-specific or specific cholinergic agonists (in parts simultaneously with selective antagonists) in anesthetized animals. Discrepancies could possibly arise from differences in anesthesia regime or depth of anesthesia, particularly in light of the known state dependency of cholinergic activation (Harris and Thiele, 2011). Moreover, the studies conducted in macaques do not explicitly dissect the role of muscarinic vs. nicotinic receptors (mAChR and nAChR, respectively) on excitability or contrast sensitivity, which requires selective blockade of these.

**Figure 1:**
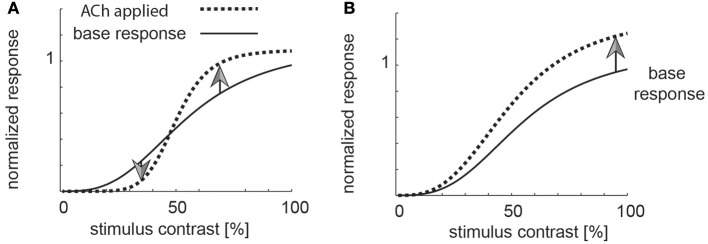
Potential role of cholinergic neuromodulation. **(A)** If acetylcholine was involved in a form a saliency enhancement, the prediction would be that weak stimuli (low contrast) are suppressed by high levels of acetylcholine, while strong stimuli would be enhanced. **(B)** If acetylcholine was involved in sensitivity enhancement, then all stimuli (weak and strong) would be processed more efficiently.

The conflicting results between Disney et al. (2012) and Soma et al. (2012) also have fundamentally different implication regarding the function cholinergic activation serves in V1. ACh induced increases of sensitivity in thalamocortical recipient layers, combined with muscarinic induced response reduction outside layer 4, could result in enhancement of strong stimuli, and filtering out of weak stimuli, i.e., a mechanism that helps saliency detection akin to the proposed roles of noradrenaline (Figure 1A for a cartoon, Aston-Jones and Cohen, 2005). Moreover, this scenario could also indicate that ACh is involved in contrast or other forms of normalization (Heeger, 1992; Carandini et al., 1997; Lee and Maunsell, 2009; Reynolds and Heeger, 2009; Carandini and Heeger, 2012; Ni et al., 2012; Ray et al., 2013; Sanayei et al., 2015). Conversely, the effects seen in layer 4, in conjunction with muscarinic induced sensitivity increase outside layer 4, would cause an increase in neuronal gain and thus an overall sensitivity enhancement (Figure 1B for a cartoon).

To address these issues, we investigated the effects of muscarinic and nicotinic receptor blockade (and by proxy their activation) on V1 neurons in awake animals. By fitting a variety of different models to our data, and performing model evaluation, we aimed to gain insight into the mechanisms by which these receptors contribute to contrast sensitivity, namely whether they best support a signal enhancement role of ACh or a saliency filtering role. We recorded contrast response functions in area V1 in macaque monkeys, while they performed a passive fixation task under control conditions and while scopolamine (muscarinic blocker) or mecamylamine (nicotinic blocker) were iontophoretically applied in the immediate vicinity of the neurons under study.

## Methods

Three male rhesus monkeys (male 5–8 years old) were used for the electrophysiological recordings reported in this study. After initial training, monkeys were implanted with a head holder and recording chambers above V1 under general anesthesia and sterile conditions (for details of surgical procedures, post-surgical analgesics and general post-surgical treatment see Thiele et al., 2006). All procedures complied with the European Communities Council Directive RL 2010/63/EC, the US National Institutes of Health Guidelines for the Care and Use of Animals for Experimental Procedures, and the UK Animals Scientific Procedures Act. They were approved by the Newcastle University AWERB committee.

### Electrophysiological recordings and drug application

The details of our iontophoretic approaches have been published before (Herrero et al., 2008). They are repeated here for ease of access.

A tungsten-in-glass electrode flanked by two pipettes was used for the recordings (Thiele et al., 2006). Drugs were applied iontophoretically through these pipettes (NeuroPhore BH-2, Digitimer, Welwyn Garden City, Hertfordshire, England). Pipette opening diameter varied between 1 and 4 μm. Pipette resistance varied between 12 and 150 MΩ, with most recordings at 20–80 MΩ.

Pipette-electrode combinations were inserted into V1 through the dura on a daily basis without guide tubes. The electrode and the pipette integrity was checked by visual inspection under the microscope before and after the recording sessions, and by measurements of the pipette impedance before and after the recording at each recording site. Drug application was continuous during blocks of “drug applied.” The duration of each block could vary depending on the number of repetitions for each condition that we aimed for, and depending on the number of eye fixation errors that the monkey made. On average drug application for each block was ~7–12 min. For the data analysis we removed the first 2 trials of each condition (8 contrasts and 2 drug states) from the data set (i.e., the first 32 trials of each block) as drug effects and recovery usually occur with a slight delay. Drugs (SIGMA Aldrich) were dissolved in distilled water. The details regarding drug concentration, pH, and application current were: scopolamine (0.1 M, pH 4.5, median current strength: 30 nA, 25 percentile: 15 nA, 75 percentile: 45 nA), and mecamylamine (0.1 M, pH 4.5, median current strength: 10 nA, 25 percentile: 15 nA, 75 percentile: 5 nA).

In all experiments we regularly compensated for the change in current during the ejection condition by increasing the hold current of one of the two pipettes, thereby keeping the overall current identical between the “hold” and “eject” conditions. This ensured that overall current level between “hold” and “eject” were identical, and therefore none of the effects described in the paper can be due to direct current effects. In addition we performed “sham” injections at a few sites in V1, by filling the barrels with saline of identical pH (4.5) and recording the neuronal activity under “hold” and “eject” conditions. None of these sham injections resulted in any significant changes to the neuronal activity.

### Data collection

Stimulus presentation and behavioral control was managed by Remote Cortex 5.95 (Laboratory of Neuropsychology, National Institute for Mental Health, Bethesda, MD, https://www.nimh.nih.gov/labs-at-nimh/research-areas/clinics-and-labs/ln/shn/software-projects.shtml). Neuronal data were collected by Cheetah data acquisition (Neuralynx, Bozeman, MT) interlinked with Remote Cortex. The waveforms of all spikes that exceeded a threshold set by the experimenter were sampled at 32 kHz. Spike data from the recording electrode were obtained by band-pass filtering the raw signal from 600–9000 Hz. To obtain single unit data, offline sorting of these spike samples was carried out based on waveform features (Neuralynx spike sorting software and custom Matlab scripts).

### Behavioral task and stimuli

#### Display

All stimuli were presented against a gray background (21 cd/m^2^) on a 20 inch analog cathode ray tube (CRT) monitor (100 Hz; 1,600 × 1,200 pixels; 57 cm from the animal). Eye position was recorded with an infrared-based camera system (Thomas Recording) and sampled at a rate of 250 Hz. The maximum luminance for 64% contrast stimuli was 34.45 cd/m^2^, while the minimum luminance was 7.55 cd/m^2^.

#### RF mapping

At the beginning of each recording receptive fields were mapped using a reverse correlation technique described previously (Gieselmann and Thiele, 2008). The RFs recorded had an eccentricity of 3–6°, with the majority at ~4.0°.

#### Contrast response functions

A trial started as soon as the monkey's eye position was within a fixation window centered on the fixation point. Following a 500 ms pre-stimulus period, a Gabor grating was presented for 700 ms, centered on the neuron's RF. The orientation and spatial frequency of the Gabor matched the neuron's orientation and spatial frequency preference, as determined by a reverse correlation technique (Gieselmann and Thiele, 2008). The Gabor moved within a Gaussian aperture at 4 Hz temporal frequency. The motion was perpendicular to the Gabor's orientation, and reversed direction at a frequency of 4 Hz (~3 time reversals during the 700 ms presentation). The contrast of the Gabor was varied between 0, 4, 8, 12, 16, 24, 32, and 64% (Michelson contrast). Following stimulus presentation the fixation point disappeared and monkeys were rewarded if their eye position had been within the fixation window (see below) for the trial duration. Twenty trials per stimulus, contrast, and drug application condition were recorded in most recordings. Cells were excluded if fewer than 10 trials per stimulus (contrast), and drug application conditions were available.

For all recordings eye movements were recorded by an infra-red based system (Thomas Recording, temporal resolution 220 Hz, spatial resolution 2.5′). Eye position during all trials was restricted to be within ±0.5–0.7° of the fixation point.

### Data analysis

First, we analyzed activity in trials before a drug was applied (i.e., trials during the baseline period). In the context of this paper we used the response period from 200 to 700 ms after stimulus onset for analysis because of our interest in the steady-state response. However, we confirmed the generality of our findings by also analyzing the responses in the period form 50–200 ms after stimulus onset. Using this window gave qualitatively identical results to those reported for the 200–700 ms response window. Stability of recording (i.e., absence of slow drifts over time) was assessed by calculating a correlation between firing rates (separately for the no drug and the drug conditions) against trial number. Neurons were included if the correlation for all 16 conditions (8 contrasts ^*^2 drug states) was not significant (*p* > 0.05/16 = 0.003125; assuming non-significant changes over time after correction for multiple comparison). A total of 74 cells investigated with and without scopolamine application passed this assessment, and 41 cells investigated with and without mecamylamine application passed this assessment.

**Figure 2:**
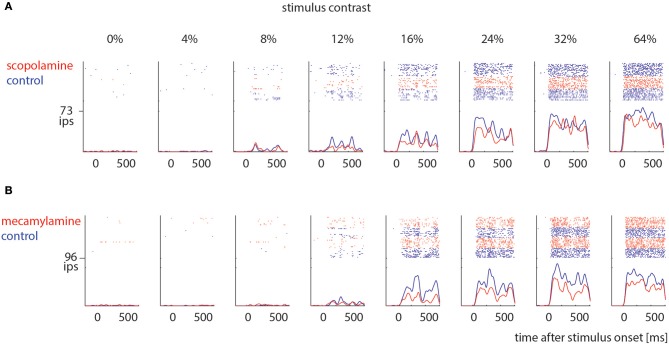
Examples of drug effects on neuronal activity across different stimulus contrast. **(A)** Cell that was measured under control conditions (blue) and when scopolamine was locally applied (red). **(B)** Cell that was measured under control conditions (blue) and when mecamylamine was locally applied (red). Ips: impulses per second.

**Figure 3:**
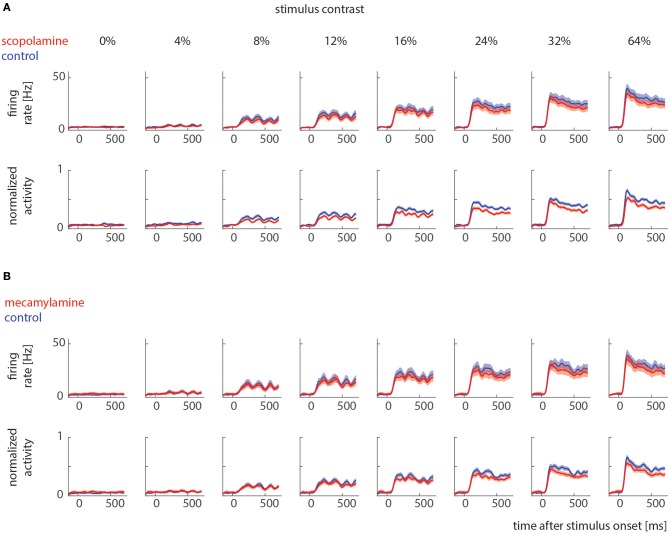
Raw and normalized population histograms of drug effects on neuronal activity across different stimulus contrast. **(A)** Population activity under control conditions (blue) and when scopolamine was locally applied (red). Upper row shows non-normalized population activity, lower row shows normalized population activity (normalized to maximum for each cell). **(B)** Population activity under control conditions (blue) and when mecamylamine was locally applied (red). Upper row shows non-normalized population activity, lower row shows normalized (for each cell) population activity. Solid lines show mean activities, shaded areas S.E.M (which sometimes was too small to be visible).

Sorted spikes were plotted for each contrast aligned to stimulus onset, and peri-stimulus time histograms (PSTH) were calculated for each condition using 1 ms bin resolution, whereby each spike was convolved with a 20 ms Gaussian envelope for smoothing (see Figure 2 for examples). These plots were visually inspected as an additional control for recording stability (in addition to the quantitative drift measure analysis described above). Single cell histograms were used to compile population histograms (Figure 3) for the different conditions.

Mean firing rates were calculated for each cell and condition in the time window from 200 to 700 ms after stimulus onset. Spontaneous activity was calculated in a time window from −300 to 0 ms before stimulus onset.

Contrast response functions (Naka-Rushton) were calculated for each cell and drug condition by fitting the following formula.

(1) $$\begin{array}{l} R\left(C\right)=R_{\max}\;*\;\frac{c^{n}}{\left(c^{n}+{c_{50}}^{n}\right)}+M \end{array}$$

to the data, whereby the fitted parameters were: *R*_*max*_ is the saturated (maximal) response, *c50* is the contrast at which the half maximal response is reached, *n* determines the slope of the contrast response function at c50, and *M* corresponds to the baseline activity. The model has been successfully used to describe contrast response functions in monkey visual cortex (Albrecht and Hamilton, 1982; Thiele et al., 2000; Williford and Maunsell, 2006). We used non-linear minimization to minimize the summed squared difference between data and model (fminsearch, Matlab 7.1, Mathworks), whereby we constrained the M to be ≥0, c50 to be in the interval [2 50], n to be in the interval [0.1 10], and Rmax to be smaller than 1.5 times the maximum response measured for a given cell. To determine whether any of the fitted parameters differed for the non-drug and the drug condition, we fitted each function independently with the Naka-Rushton function.

### Effect of cholinergic modulation on neurometric functions

We quantified the effect of cholinergic modulation on neuronal sensitivity by calculating neurometric functions for each cell under control and drug applied conditions. This was done by calculating the Area Under the Receiver Operating Curve (AUROC) for each contrast, by comparing it to the 0% contrast condition (time window for analysis: 200–700 ms after stimulus onset). This yields an AUROC value of 0.5 for the 0% contrast condition, and values that can differ from 0.5 for all other contrast conditions. We fitted these AUROC data with a Weibull function (maximum likelihood estimation):

(2) $$\begin{array}{l} AUROC=1-\left({0.5\;*\;e}^{{\left(\frac{c}{\alpha }\right)}^{\beta }}\right) \end{array}$$

where “AUROC” corresponds to AUROC value measure, or the probability correct of an ideal observer performance, “c” is stimulus contrast, “α” corresponds to contrast threshold (~82% ideal observer performance) and “β” corresponds to the slope of the function at threshold. We fitted this function separately to the two data sets (control and drug applied, respectively), and also fitted a single function to the joint data set, i.e., forcing α and β to attain the same value for the two conditions. This yielded separate goodness of Weibull fits (χ^2^) and a single goodness of Weibull fit (χ^2^). If the joint fit was as good as the fit to the separate data sets (i.e., if the χ^2^ for the common fit was not significantly different from the χ^2^ for separate data sets, χ^2^ distribution; degrees freedom = 2; *p* > 0.05), the thresholds for control and the drug condition were deemed indistinguishable. By contrast, if a common fit was worse than separate fits (*p* < 0.05), thresholds for the two conditions were considered significantly different (Watson, 1979; Thiele et al., 2000).

### Model evaluation

To determine whether the effects of cholinergic modulation are best captured by a response gain model, a contrast gain model, an additive model, or normalization models the control and drug data were jointly fit with the following equations:

Response gain model:

(3) $$\begin{array}{l} \text{Control Condition}:\;R\left(C\right)=R_{\max}\;*\;\frac{c^{n}}{\left(c^{n}+{c_{50}}^{n}\right)}+M \end{array}$$

$$\begin{array}{l} \text{Drug Condition}:\;R\left(C\right)={drug\;*\;R}_{\max}\;*\;\frac{c^{n}}{\left(c^{n}+{c_{50}}^{n}\right)}+M \end{array}$$

Contrast gain model:

(4) $$\begin{array}{l} \text{Control Condition}:\;R\left(C\right)=R_{\max}\;*\;\frac{c^{n}}{\left(c^{n}+{c_{50}}^{n}\right)}+M \end{array}$$

$$\begin{array}{l} \text{Drug Condition}:\;R\left(C\right)=R_{\max}\;*\;\frac{c^{n}}{\left(c^{n}+{{drug\;*\;c}_{50}}^{n}\right)}+M \end{array}$$

Additive model:

(5) $$\begin{array}{l} \text{Control Condition}:\;R\left(C\right)=R_{\max}\;*\;\frac{c^{n}}{\left(c^{n}+{c_{50}}^{n}\right)}+M \end{array}$$

$$\begin{array}{l} \text{Drug Condition}:\;R\left(C\right)=R_{\max}\;*\;\frac{c^{n}}{\left(c^{n}+{c_{50}}^{n}\right)}+drug\;*\;M \end{array}$$

Normalization model:

$$\begin{array}{l} \text{Control Condition}:\;R\left(C\right)=R_{\max}\;*\;\frac{c^{n}}{\left(c^{n}+{c_{50}}^{n}\right)}+M \end{array}$$

(6) $$\begin{array}{l} \text{Drug Condition}:\;R\left(C\right)={drug*\;R}_{\max}\\ \;*\;\frac{c^{n}}{\left(drug\;*\;c^{n}+{c_{50}}^{n}\right)}+M \end{array}$$

Saliency filter (slope) model:

(7) $$\begin{array}{l} \text{Control Condition}:\;R\left(C\right)=R_{\max}\;*\;\frac{c^{n}}{\left(c^{n}+{c_{50}}^{n}\right)}+M \end{array}$$

$$\begin{array}{l} \text{Drug Condition}:\;R\left(C\right)=R_{\max}\;*\;\frac{c^{drug\;*\;n}}{\left(c^{drug{\;}^{*}\;n}+{c_{50}}^{drug\;*\;n}\right)}+M \end{array}$$

Where the different parameters were as described in equation 1, except for the additional parameter “drug.” This single additional free parameter occurs for the drug applied condition, and it can affect the contrast dependent responses. Note that all models have exactly the same number of free parameters. To determine which model best described the effect on a given cell, we calculated the χ^2^-error for each of these functions and performed model evaluation by Akaike's information criterion (AIC, Burnham and Anderson, 2004):

(8) $$\begin{array}{l} AIC=\;\chi^{2}+2k \end{array}$$

where k corresponds to the number of free parameters in the model, i.e., 5 respectively. We thus obtained AIC1 to AIC5 for each cells, and calculated Akaike weights (w_i_) for final model comparison:.

(9) $$\begin{array}{l} wi=\frac{e\left(-\frac{\delta_{i}}{2}\right)}{\sum_{n=1}^{5}e\left(-\frac{\delta_{n}}{2}\right)} \end{array}$$

Here, δ_i_ corresponded to the AIC_i_-AIC_min_, where AIC_min_ was the smallest AIC obtained for the 5 model fits. The larger the w_i_, the more evidence existed in favor of model i.

Additionally we calculated how much variance each model accounted for (Carandini et al., 1997; Roberts et al., 2005, 2007), and used this as an additional measure to assess which model best described the data overall.

## Results

We recorded contrast tuning curves for 74 cells under control conditions and when scopolamine was applied, and for 41 cells under control conditions and when mecamylamine was applied. Figure 2 shows responses from 2 example cells. For both cells the activity increased with increasing contrast (*p* < 0.001, 2 factor ANOVA), and drug application reduced neuronal activity (*p* < 0.001, 2 factor ANOVA). These cells exemplify the pattern found across our neuronal populations (Figure 3).

**Figure 4:**
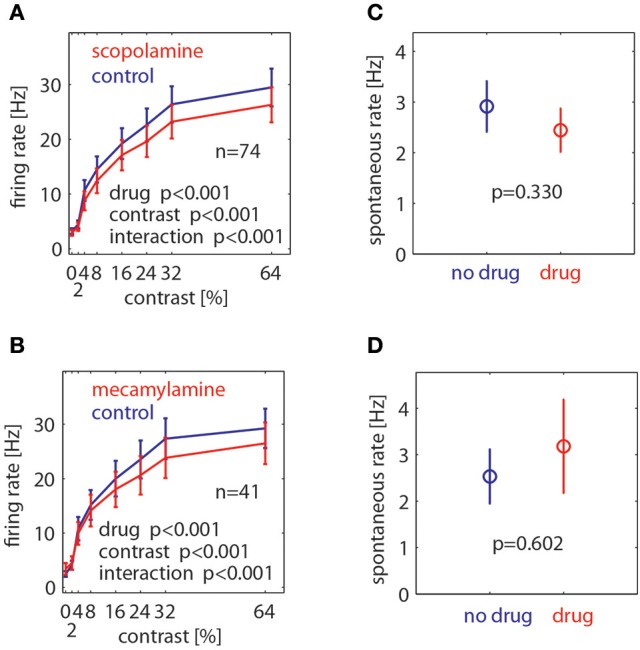
Effect of muscarinic and nicotinic receptor blockade on neuronal firing rates at different stimulus contrasts and for spontaneous activity. **(A)** Effect of muscarinic receptor blockade (scopolamine application) on neuronal firing rates at different Gabor contrasts. **(B)** Effect of nicotinic receptor blockade (mecamylamine) on neuronal firing rates at different Gabor contrasts. **(C)** Effect of muscarinic receptor blockade on spontaneous activity. **(D)** Effect of nicotinic receptor blockade on spontaneous activity. Blue symbols show mean ± S.E.M of control activity, red symbols show mean ± S.E.M of drug applied activity. *P*-values indicate significance, number of cells recorded is indicated by the insets “n.”

To assess the drug effects quantitatively we calculated the mean rate for each cell in the time window from 200 to 700 ms after stimulus onset for each contrast and drug condition, and also for the pre-stimulus period from −300 to 0 ms. These data are shown in Figure 4. We calculated a 2 factor repeated measures (RM) ANOVA (factor1: contrast, factor 2: drug application) to determine whether contrast and/or drug significantly affected firing rates, and whether there was a significant interaction between these. Drug effects on the pre-stimulus period were assessed with a paired *t*-test. As expected, contrast significantly affected firing rates (*p* < 0.001, 2 factors RM ANOVA). Moreover, muscarinic as well as nicotinic blockade significantly affected firing rates (*p* < 0.001, 2 factors RM ANOVA). In both cases we found a significant interaction between contrast and drug application. Figure 4 shows that drug induced reduction was larger for higher firing rates (higher contrast). Neither drug affected spontaneous activity (scopolamine: *p* = 0.33, mecamylamine: *p* = 0.602, paired *t*-test).

**Figure 5:**
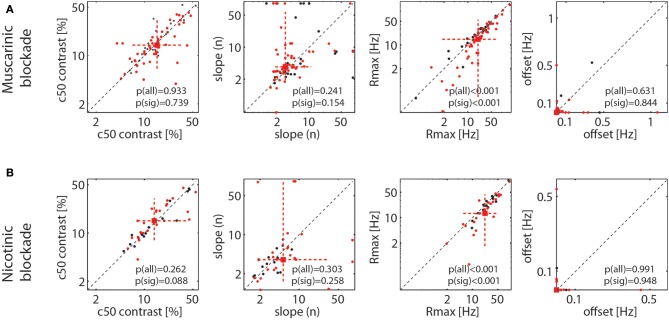
Effect of muscarinic and nicotinic receptor blockade on parameter values of the fitted contrast response function (NAKA-Rushton) for the control and the drug applied conditions. **(A)** Effect of muscarinic receptor blockade on the contrast at the half maximal response, (c50), the slope of the function (n), the maximal response (Rmax), and the response offset (estimated spontaneous activity). **(B)** Same as in **(A)**, but for nicotinic receptor blockade. Black symbols show the fitted parameter values for all recorded cells, red symbols for cells where drug application had a significant effect on their firing rate. Red square symbol shows the median of the parameter estimate for cells significantly affected by drug application, dashed red lines show 10 and 90% percentiles. *P*-values indicate whether parameter estimates differ significantly between control and drug applied conditions when all cells were taken into account [p(all)] and when only cells significantly affected by the drug application were taken into account [p(sig)].

As stated, Figure 4 shows that drug induced reduction was larger for higher firing rates (higher contrast), which suggests that drug application mostly resulted in a proportional scaling of the contrast response function, rather than a shift to either the left or right, or a change in the overall slope. To quantify this across the population of cells, we fitted each cell's responses with a Naka-Rushton function (methods). This allows determining whether the maximum response (Rmax), the location of the half maximal response (c50), the slope of the contrast response function (n), or the offset (M) was affected by drug application. The results are shown in Figure 5. It shows that the only parameter of the Naka-Rushton function significantly affected by drug application was the maximal response (Rmax), which was reduced when either muscarinic receptors (Figure 5A), or nicotinic receptors were blocked (Figure 5B). The results held also, when only cells significantly affected by drug application were included in the analysis [see p(sig) insets in Figure 5], although there was a trend for a significant change of the c50 location (an increase in c50) when nicotinic receptors were blocked (*p* = 0.088), provided significantly affected cells were analyzed.

### Neuronal discrimination abilities

**Figure 6:**
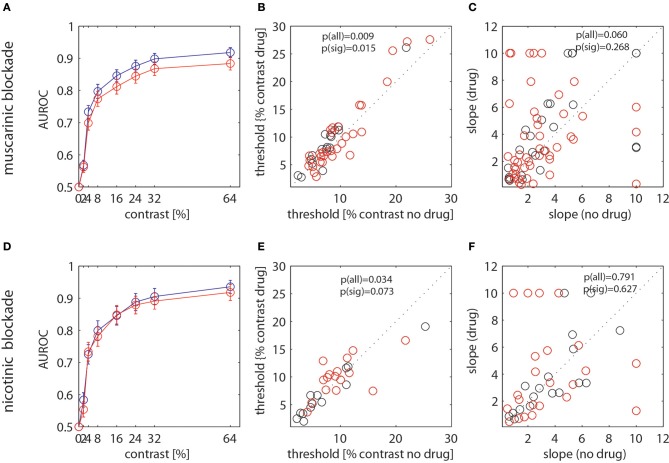
Analysis of drug effects on neurometric functions. **(A,D)** AUROC values for the control (blue) and the drug (red) condition when scopolamine was applied. **(B,E)** Neuronal thresholds derived from the Weibull fits to the AUROC values for the control (abscissa). **(C,F)** Slope of the Weibull function at the threshold location. **(A–C)** show data when scopolamine was applied, **(D–F)** show data when mecamylamine was applied. Red dots show data were separate fits of the Weibull function yielded significantly better fits to the data than a single combined fit to the two data sets. Black dots show those were a single fit was not significantly worse than separate fits. *P*-value insets indicate whether the thresholds (slopes) derived from separate fits differed significantly for the control and the drug conditions (Wilcoxon signed rank test). P(all) indicates the comparison when all cells were taken into account, p(sig) show the comparison for cells that were significantly better fitted with separate functions.

While the previous analyses reveal that nicotinic and muscarinic receptor blockade affect neuronal contrast response functions, they do not yield insight into how this would affect discrimination abilities. For example changes in Rmax or c50 on their own do not provide information about sensitivity *per se*, even though they may hint that these would be affected. This is because the half maximal response of a neuron does not relate in a meaningful to neuronal discriminability, as it ignores e.g. maximum firing rate in relation to baseline firing rate and rate variability. For example, if drug induced changes were minimal or absent at relatively low contrasts, neuronal discrimination (detection) abilities might still be unaffected. To quantify neuronal discrimination/detection abilities we calculated AUROCs and fitted Weibull functions to the drug applied and the drug not applied data (see methods). We thus obtained neurometric functions (Britten et al., 1992; Thiele et al., 2000) for each cell for the different drug conditions. From these fits we obtained estimates of neuronal sensitivity (neuronal thresholds) and of the slope of the Weibull function at threshold. The results are shown in Figure 6.

AUROC values were (largely) lower when the drugs were applied for both, the muscarinic and the nicotinic blockade. The effects overall appeared larger when muscarinic receptors were blocked than when nicotinic receptors were blocked. Thresholds of the Weibull function fits are defined as the ability of an ideal observer to detect the stimulus with an accuracy of 82%. Median thresholds were 9.62% and 11.5% contrast for the control and the scopolamine applied conditions. They were 10.0% and 10.7% contrast for the control and the mecamylamine applied condition. Blockade of muscarinic and nicotinic receptors resulted in significantly increased thresholds when considering the entire sample of cells, i.e., reduced neuronal sensitivity (see *p*-value insets in Figures 6B,E, Wilcoxon sign rank test). Considering only those cells where separate fits yielded significantly better fits than a joint fit, the thresholds were significantly increased when muscarinic receptors were blocked, and a trend toward a significant increase was observed when nicotinic receptors were blocked. The slope at threshold was not significantly affected by drug application for either of the conditions, although there was a trend for increased slopes when all cells were taken into account for the muscarinic blockade condition (Figure 6C).

### Model evaluation

The data shown in Figure 5 do not address the question whether the effects of drug application are best explained by response gain, contrast gain, additive, normalization models, or slope gain models. To address this quantitatively, we fitted each cell's responses with response gain, contrast gain, additive gain, a normalization and a slope gain model. We calculated AIC weights and variance accounted for, for each model to determine which of the models yielded the best model fit for a given cell. The largest AIC weight indicates which model has the best evidence (across the models applied). Moreover, the largest average variance accounted for gives an indication which model best fits that majority of cells. In this analysis we focus on cells where drug application had a significant effect on neuronal activity. The reason for this is that cells not affected by drug application, should in principle be equally well fit by any of the models as the drug parameter (see methods) should be 1 in that case, which would make all the models identical.

**Figure 7:**
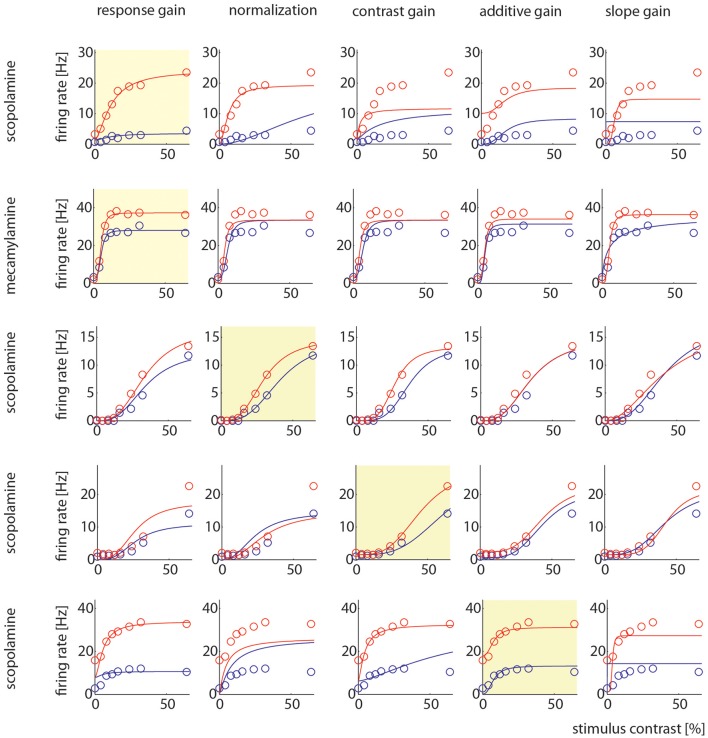
Model comparison for example cells. Each row shows how well the 5 different models could be fitted to the control and drug applied activity levels. The yellow insets highlight which particular model yielded the largest AIC weight. The different models are named at the top, the different drugs used in the particular recording are indicated on the right.

Example cells where one model gave better fits than other models are shown in Figure 7. It shows two examples where the response gain yielded the best fits, on example where the normalization model yielded the best fit, one example where contrast gain, and one example where the additive gain model yielded the best fit.

**Figure 8:**
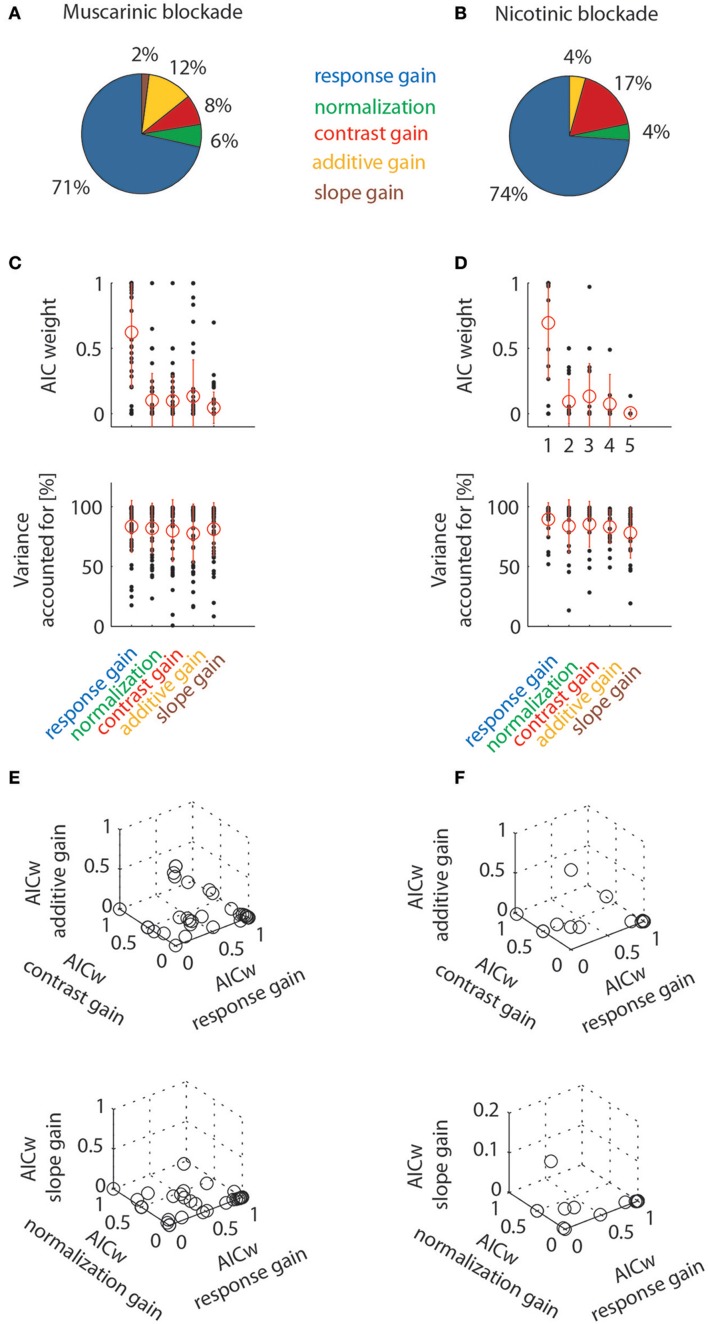
Model comparison at the population level by means of Aikake information criterion weights (AICw) and variance accounted for. Left column shows data for cells tested with muscarinic blockade, right column shows data for cells tested with nicotinic blockade. Only cell significantly affected by drug application were taken into account. **(A,B)** Percentage of cells where drug effects were best fit by one of the models employed (quantified by taking the largest AIC weights). **(C,D)** Distribution of AIC weights and variance accounted for, given the different models. Red symbols denote means and standard deviation. **(E,F)** Triplet wise model comparison that shows how AIC weights are linked for individual cells. Most data points are clustered towards high values for response gain models (they are often overlayed on top of one another, which makes the crowding towards that location less apparent than really present).

The percentages of cells best fit by the different models are shown in Figure 8. When muscarinic receptors were blocked, most of the cells were best fit by a response gain model (71%), followed by additive gain, contrast gain, normalization, and slope models. The percentage of the latter gain models was comparatively small (12, 8, 6, and 2%, respectively). A similar picture emerges when analyzing the data for nicotinic blockade. The majority of cells from this data set were equally best fit by a response gain model (74%), followed by the contrast gain (17%), additive and normalization gain models (4% each). The slope gain model only ever best described the data in 1/66 significantly affected cells across both drug conditions. The best fit counts (number of cells) for the different models were not different between the two drug conditions [χ^2^ = 1.717, *df* = 4, χ^2^/*df* = 0.43, *P*_(χ2>1.717)_ = 0.7876)]. Figure 8 additionally shows the distributions of AIC weights, and the variance accounted for, across the models. The response gain models on average had larger AIC weights than the other models. This is the case even though all models yielded fairly good fits to the data (as evidenced by the fairly large values of variance accounted for, see also the examples in Figure 7). Finally, triplet wise AIC weight comparisons are shown between different models in Figure 8 (bottom rows). These plots show that the majority of AIC weights are clustered toward large values (close to 1) for the response gain models. Thus, response gain models best describe the action of ACh for the majority of cells in macaque area V1. Nicotinic and muscarinic receptor stimulation would increase response gain.

## Discussion

Here we investigated the effects of muscarinic and nicotinic blockade on V1 contrast responses. Both manipulations reduced neuronal responses and reduced neuronal sensitivity. Fitting the data with different gain models indicates that nicotinic receptors and muscarinic receptors mostly affect contrast sensitivity by response gain changes.

In the following discussion we will mostly focus on data derived from *in vivo* studies. Many previous studies used indirect measures to assess the contribution of mAChRs or nAChR to neuronal sensitivity. They often applied non-selective agonists either alone or in conjunction with nAChR or mAChR antagonists. The reduction or absence of effects when antagonists were co-applied served as an indicator that effects seen were receptor selective. We will discuss these studies as if the receptors had been selectively activated or suppressed, and thereby follow the preferred interpretations given by the respective authors.

### Basic effects of nicotinic manipulation

Most studies have reported that increased nicotinic drive in cortical areas results in increased stimulus driven activity. No controversy exists in this respect for layer IV (Disney et al., 2007; Bhattacharyya et al., 2012; Soma et al., 2012). However, outside layer IV, Disney et al. (2007) reported that nicotinic activation resulted in reduced neuronal activity, while Soma et al. (2012) reported increased activity across all cortical layers (even though they argued that the main facilitation was mediated by muscarinic effects, see below). Bhattacharyya et al. (2012) equally found overall increased responses across all cortical layers upon nicotinic and muscarinic stimulation in V1 of the tree shrew, In a previous study, we found that nicotinic blockade caused overall reduced activity in macaque V1 (Herrero et al., 2008), which matches our results reported here. By inference, we would predict that nicotinic activation would result in increased activity. Neither our previous study, nor our current study could assign recording sites to specific cortical layers, due to the nature of day-to-day sampling of neurons in chronically implanted task performing macaques. However, it is unlikely that our recordings were predominantly in layer IV. Rather the daily access makes it likely that a large proportion of neurons were recorded from layers II and III. This assumption is based on the following. The electrode would first traverse layers I, II, and III. Recording normally commenced once good stimulus driven spiking activity was obtained, as would be typical for layers II and III. Given that a single recording required a fairly large number of trials, we usually recorded only from 1 to 2 sites per day before the animals stopped working. Thus, if our data are largely from layer II and III, they would be in line with the results by Soma et al. (2012) and Bhattacharyya et al. (2012), and less with those reported by Disney et al. (2007, 2012).

### Basic effects of muscarinic manipulation

As is the case for nAChR manipulation, discrepant results have been reported for the effects of mAChR on neuronal excitability in V1. Herrero et al. (2008) found an overall reduction of neuronal activity in V1 when mAChRs were blocked by scopolamine, along with a reduction in attentional modulation of V1 activity. Disney et al. (2007, 2012) found that outside layer IV ACh application generally reduced neuronal activity. This reduction was mediated by increased activity in inhibitory cells. Based on the distribution of mAChRs and nAChRs they inferred that the reduction was largely mediated by mAChRs (Disney et al., 2012). Conversely, Soma et al. (2012) found that cholinergic induced facilitation of activity in V1 was mediated largely through muscarinic activation (with some nicotinic contribution). Additionally, the small number of cells inhibited by ACh, were also affected through muscarinic mechanisms. Bhattacharyya et al. (2012) reported overall increased baseline and stimulus induced activity across cortical layer (even if less so in layer 4) upon muscarinic activation. Application of scopolamine resulted in overall reduced neuronal activity in our data set, which would be in line with the results reported by Soma et al. (2012) and Bhattacharyya et al. (2012), but not by Disney et al. (2012).

It is unclear how the discrepancy between studies arises. It has been suggested that differences in anesthesia regimes might contribute, but this can be ruled out for our study as it was performed in awake task performing (passive fixation) monkeys. Moreover, it could be due to differences in the dosages applied, as Disney et al. (2012) reported differences when comparing low vs. high ACh application currents. The currents used in our study were relatively low, but since we used selective antagonists, rather than agonists, direct current comparison is not possible. However, Herrero et al. (2008) also applied ACh, and the currents used in their study were usually on the low end of those applied by Disney et al. (2012), where Disney et al found ACh induced suppression. Herrero et al. (2008) reported ACh induced activity increases. It is thus unlikely that the discrepancies between the two studies can be explained by a switch from suppression to facilitation once high levels of ACh were applied (as reported by Disney et al., 2012). However, it is still possible that baseline ACh levels differ strongly between anesthetized and awake animals, and thus switches from facilitation to inhibition occur at different application levels. Despite this, the overwhelming number of studies reported that ACh application results on increased activity in V1 (Sillito and Kemp, 1983; Sillito et al., 1985; Sato et al., 1987; Roberts et al., 2005; Zinke et al., 2006; Herrero et al., 2008; Goard and Dan, 2009; Kuo et al., 2009; Rodriguez et al., 2010; Soma et al., 2012, 2013; Pinto et al., 2013), and in other cortical areas (McKenna et al., 1988; Metherate et al., 1988a,b; Matsumura et al., 1990; Tremblay et al., 1990; Bhattacharyya et al., 2012, 2013; Thiele et al., 2012; Yang et al., 2013; Major et al., 2015; Sun et al., 2017).

It is remains puzzling how an overall facilitation occurs given that nicotinic and muscarinic somatic receptors reside predominantly on inhibitory interneurons (Disney et al., 2006, 2014; Disney and Aoki, 2008; Disney and Reynolds, 2014). ACh application should result in increased gabaergic drive and thus reduced activity, as found by Disney et al. (2012). However, it could be the case that the cortex can adjust its operational regime to be always in a state of balanced excitation and inhibition. Thus, changes to one component automatically result in changes to the other component, through compensation mechanisms. Such an increase in balanced excitation and inhibition can result in overall increased excitability, which is manifests are changes in response gain. It thereby also enhances coding abilities (Shew et al., 2011).

### Cholinergic modulation of contrast sensitivity

We found that nicotinic as well as muscarinic blockade reduced neuronal contrast sensitivity and the Rmax values of the contrast response function, without (or with very little) effect on the contrast at half maximum response (c50). This is similar to the effects reported in previous studies, where non-specific cholinergic agonist application resulted in changed Rmax values, and little (or no) change to the c50 location (Disney et al., 2012; Soma et al., 2012). Muscarinic or nicotinic activation in V1 of the tree shrew also mostly affected Rmax and baseline responses, with no systematic effect on c50 (Bhattacharyya et al., 2012). Despite this overall report, Disney et al. (2007) data appear to show an increase in c50 with nicotinic stimulation (see their Figure 8). In our data, nicotinic blockade (rather than stimulation) resulted in a trend toward increased c50 values, provided only neurons significantly affected by drug application were taken into account. This would be the opposite of what Disney et al.'s Figure 8 shows. Small increases in c50 with acetylcholine application were also found in rat primary visual cortex, although there, as in primate V1, the main change occurred in Rmax (Soma et al., 2013). Stimulation of tree shrew basal forebrain (BF) resulted in reduced c50 and increased Rmax values (Bhattacharyya et al., 2013). This would be largely compatible with the data presented here, if BF stimulation exclusively activated cholinergic cells. However, this is not the case (Henny and Jones, 2008) and as such it is difficult to directly compare data sets. The reduction in c50 in their data set upon BF activation is much more pronounced than any changes seen in our data, or the data of other studies directly manipulating cholinergic activation in V1. This suggests that the c50 changes were largely due to long range gabaergic influences (or a combination of gabaergic and cholinergic effects) resulting from BF activation.

The changes seen in neuronal activity and those seen in Rmax were often interpreted as improving neuronal sensitivity, but most studies did not quantitatively assess the changes. Disney et al. (2007) assessed changes in sensitivity by finding the point on the contrast response function were responses were reliably outside the response range of spontaneous activity. Assessed in this way, they found that ACh application improved contrast sensitivity for layer IV neurons. It is not clear how this would translate to behavioral measures. To do the latter, previous studies have compared psychometric and neurometric functions (e.g., Vogels and Orban, 1990; Britten et al., 1992; Thiele et al., 2000, 2001; Luna et al., 2005; Palmer et al., 2007). In our study, psychometric functions were not available, but neurometric function comparison showed that muscarinic and nicotinic blockade reduced neuronal sensitivity, although changes of the median sensitivities were relatively modest, even if significant across the population.

### Mechanisms affected by nicotinic and muscarinic manipulations

Most of the above cited studies interpreted the changes seen in Rmax, and the absence of consistent changes in c50 upon cholinergic manipulation to be a sign of altered neuronal response gain (McAdams and Maunsell, 1999; Williford and Maunsell, 2006) rather than contrasts gain (Reynolds et al., 2000). However, additive gain (Thiele et al., 2009), normalization mechanisms (Tolhurst and Heeger, 1997; Lee and Maunsell, 2009, 2010; Reynolds and Heeger, 2009; Carandini and Heeger, 2012; Ni et al., 2012; Sanayei et al., 2015), or a slope gain (Aston-Jones and Cohen, 2005) could also account for these effects. This has not been quantitatively addressed in any of the previous studies. To do so we fitted 5 different models to our data and used AIC weights to determine which model best described the effects seen upon nicotinic or muscarinic blockade. As expected, nicotinic receptors affect contrast sensitivity largely by altering the response gain (74% of the cells). Normalization mechanisms best accounted for the effects seen in only 4% of the data, and additive or contrast gain effects best described the effects seen in 17 and 12% of the cells. Slope gain effects best described the data in only 4% of the cells. Very similar results were found when manipulating muscarinic receptors. Thus, cholinergic modulation affects response gain in ~3/4 of the cells in V1, while slope gain effects are very rarely seen. This argues against the possibility that ACh could be involved in saliency filtering, but it is rather involved in sensitivity enhancement.

Normalization mechanisms, as employed in our modeling, best described our results in only ~5% cells. Normalization requires inhibitory interactions, and hence our data suggests that this type of inhibition is not the dominant feature of cholinergic manipulation. At the same time, the stimuli employed may not have been ideal to investigate this, as their size was largely confined to the classical receptive field. Using different sized stimuli, flanking stimuli, or cross orientation inhibition, might yield better insight into this question. However, contrast normalization is a well-established phenomenon in V1 (Carandini and Heeger, 1994, 2012; Carandini et al., 1997; Tolhurst and Heeger, 1997; Albrecht et al., 2002), and if cholinergic mechanisms were a main driver thereof we should have been able to uncover these with our models.

The predominance of response gain for nicotinic modulation is predictable based on the location of nicotinic receptors on thalamocortical terminals (Hasselmo and Bower, 1992; Gil et al., 1997; Kimura et al., 1999; Kimura, 2000; Disney et al., 2007), and their ability to proportionally scale input efficacy. Our data show that muscarinic receptors also often result in overall increased excitability, which can result in proportional scaling of inputs, and thus response gain changes.

Whether the changes induced by muscarinic modulation are exclusively a direct consequence of altered neuronal excitability, or are also shaped by alteration of recurrent interactions or feedback from higher areas by acting on pre-synaptic M2 receptors (Kimura and Baughman, 1997) is currently unknown. Increased excitability could be mediated by M1 receptor activation (McCormick and Prince, 1985; McCormick, 1989). This could increase the excitability of local networks by e.g. M-current reduction, which on its own might not result in increased neuronal activity (we did not see changes in spontaneous activity across our population), but it would enhance stimulus firing rates, once neurons receive input drive. Through activation of M2- receptors ACh would reduce the local recurrent processing (Hasselmo and Bower, 1992; Gil et al., 1997), but whether this would equally reduce feedback influences from higher areas is unknown. The latter is somewhat unlikely given that contrast sensitivity in V1 is reduced upon feedback removal (Hupé et al., 1998). Reduction of feedback influences by M2 receptor activation should thus result in reduced contrast sensitivity upon ACh stimulation, while our data argue for the opposite.

Thus, overall ACh may increases local cellular excitability, cause a decoupling of local recurrent interactions, but also improve integration of feedback signals terminating predominantly in layer 1 (Rockland and Pandya, 1979). Improved integration of feedback signals could be a result of M-current reduction. It would allow signals arriving at the apical dendrite in layer 1 to have more impact on signal integration at the axon hillock. Together these effects would alter the state of the local network, and induce changes which go beyond simple firing rate changes, thereby improving overall coding abilities (Minces et al., 2017; van Kempen et al., 2017).

## Conclusions

Muscarinic and nicotinic receptors contribute to contrast sensitivity of neurons in V1 in awake, passively fixating monkeys. Blockade of these receptors reduces the maximum response (Rmax) without affecting c50 of the contrast response function. Additionally, neuronal thresholds are reduced as assessed by neurometric function. By proxy, we predict that increasing nicotinic or muscarinic drive would increase Rmax and neuronal sensitivity, although that may depend on the characteristics of the inverted U-curve of neuromodulation (Smucny et al., 2015). Nicotinic and muscarinic modulation results predominantly in proportional scaling of response functions (response gain). Thus, the two receptors affect the local network in similar, and seemingly cooperative ways.

## Author contributions

JH recorded the data and contributed to the manuscript, MG supported data acquisition and contributed to the manuscript, AT conceived the study, analyzed the data and wrote the manuscript and made the figures.

### Conflict of interest statement

The authors declare that the research was conducted in the absence of any commercial or financial relationships that could be construed as a potential conflict of interest. The reviewer KK and handling Editor declared their shared affiliation.
